# Emerging treatments for scleroderma/systemic sclerosis

**DOI:** 10.12703/r/10-43

**Published:** 2021-05-05

**Authors:** Jane L Zhu, Samantha M Black, Henry W Chen, Heidi T Jacobe

**Affiliations:** 1 Department of Dermatology, University of Texas at Southwestern Medical Center, Dallas, TX, USA

**Keywords:** Systemic Sclerosis, Scleroderma

## Abstract

Systemic sclerosis (SSc) is a connective tissue disease characterized by progressive fibrosis of the skin and internal organs and has significant clinical sequelae. Management of SSc cutaneous disease remains challenging and often is driven by extracutaneous manifestations. Methotrexate is the typical first-line therapy for patients with early progressive cutaneous disease. However, in patients with diffuse progressive skin disease and inflammatory arthritis, methotrexate or rituximab monotherapy should be considered. First-line therapy for patients with concomitant myositis includes methotrexate or intravenous immunoglobulin (IVIG). For patients with both cutaneous findings and interstitial lung disease, studies have suggested the efficacy of mycophenolate mofetil or rituximab. Second-line therapies, including UVA-1 phototherapy, IVIG, or rituximab, can be considered in patients with disease refractory to first-line treatments. Clinical trials investigating the utility of emerging therapies such as abatacept and tocilizumab in the treatment of SSc are under way, and preliminary results are promising. Nonetheless, all patients with SSc benefit from a gentle skin-care regimen to alleviate pruritis, which is a commonly reported symptom. Additional cutaneous manifestations of SSc include telangiectasias, calcinosis cutis, microstomia, and Raynaud’s phenomenon. Telangiectasia may be managed with camouflage techniques, pulse dye laser, and intense pulse light. Calcinosis cutis therapy is guided by the size of the calcium deposits, although treatment options are limited. Mouth augmentation and oral stretching exercises are recommended for patients with reduced oral aperture. Raynaud’s phenomenon is treated with a combination of lifestyle modification and calcium channel blockers, such as amlodipine. Overall, SSc is a clinically heterogenous disease that affects multiple organ systems. Providers should assess extracutaneous involvement and use evidence-based recommendations to select the most appropriate therapy for patients with SSc.

## Introduction

Systemic sclerosis (scleroderma, SSc) is a connective tissue disease characterized by overproduction and deposition of collagen and leads to progressive fibrosis of the skin and internal organs (that is, gastrointestinal tract, heart, lungs, and kidneys). Raynaud’s phenomenon, puffy edematous digits, and the presence of antinuclear antibody (particularly hallmark antibodies) are distinguishing clinical features that should raise a clinician’s suspicion for a diagnosis of early SSc^1^.

**Figure 1.**
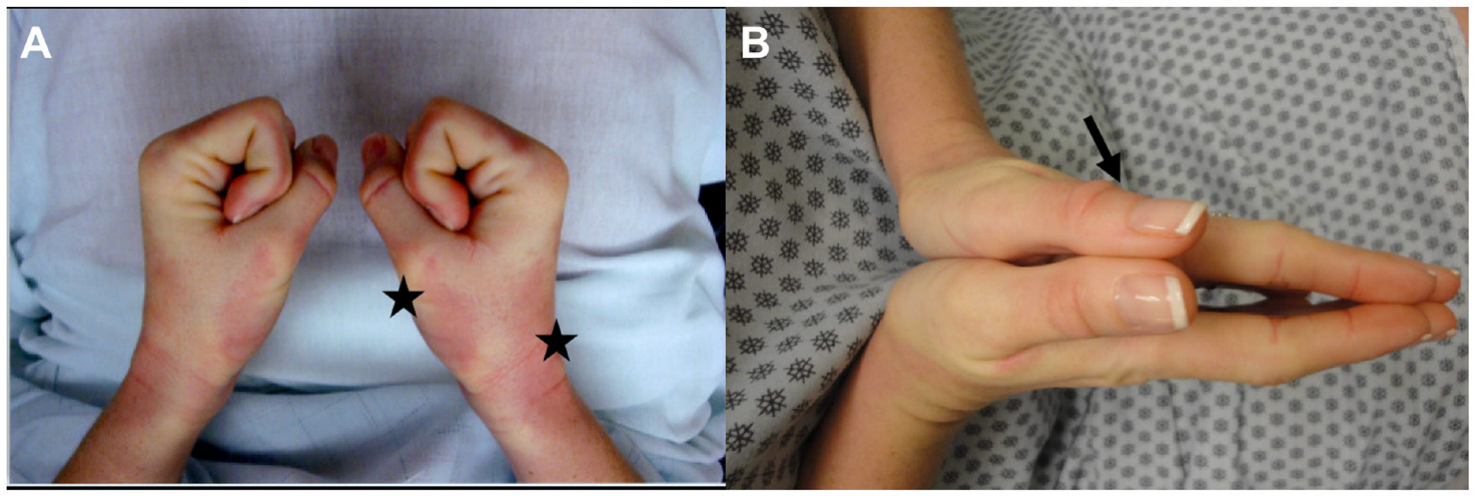
Edematous and sclerotic phases of cutaneous systemic sclerosis. (**A**) The edematous phase is characterized by non-pitting edema of the digits and erythematous indurated plaques (stars). (**B**) The sclerotic phase results when the skin is thickened, resulting in complications such as joint contractures and sclerodactyly. Note thickened skin folds (arrow).

Although the disease is multisystemic, skin involvement remains a cardinal feature and often is used to categorize patients into different subsets and assess disease prognosis. The progression of SSc cutaneous disease occurs in three stages: edematous, sclerotic, and atrophic. The earliest cutaneous manifestation of SSc typically includes non-pitting edema of the fingers (Figure 1A). The skin of affected digits, extremities, and in some cases trunk gradually thickens, resulting in complications that include joint contractures, disabling sclerodactyly, and digital ulcers from local ischemia and vascular insufficiency (Figure 1B)^2,3^. In the late stages of the disease, the affected skin is thin and atrophic. Other cutaneous manifestations include telangiectasias, calcinosis cutis, reduced oral aperture, and hypo- and hyper-pigmented areas of skin (salt and pepper pigmentary change).

SSc is classified into two main clinical phenotypes: limited cutaneous SSc (lcSSc) and diffuse cutaneous SSc (dcSSc). Patients with lcSSc have skin involvement confined to areas distal to their elbows or knees with or without involvement of the face, whereas patients with diffuse involvement have skin changes proximal to the elbows or knees with acral and truncal involvement. In addition to differing in the extent of skin involvement, patients with lcSSc and dcSSc differ in the rate at which their disease progresses. Limited SSc has an insidious onset. Although these patients ultimately are more likely to have pulmonary arterial hypertension and digital ulceration, they often have Raynaud’s phenomenon for many years prior to any other clinical manifestations and the skin involvement in the fingers can be very subtle. In contrast, patients with diffuse SSc often have rapid onset of cutaneous disease with early internal organ involvement. Furthermore, patients with diffuse cutaneous involvement have significant systemic disease burden early in their course because of severe cardiac, pulmonary, and gastrointestinal involvement; their 10-year survival rate is 65% as opposed to 92% for patients with limited cutaneous involvement^4^.

Despite the advances that have been made over the years to understand the pathogenesis of SSc, treatment remains challenging. There are no specific treatments for SSc, and disease management often is dictated by organ-specific complications^5^. In this review, we summarize current and emerging treatment options for SSc-related cutaneous disease.

## Therapeutic options for systemic sclerosis–related cutaneous disease

New trials and increased understanding of pathogenesis have produced a number of promising therapies for SSc-related skin disease (Table 1). Each of these may be considered depending on SSc subtype, status of extracutaneous organ involvement, and stage of evolution of skin disease. It is also important to differentiate cutaneous manifestations amenable to treatment with immunosuppressives, such as the inflammatory, edematous, or early stage of sclerotic skin disease versus Raynaud’s phenomenon, which are not responsive to immunosuppressives.

**Table 1.** Treatments for scleroderma by level of evidence.

Treatment	Level of evidence
UVA-1	2
Methotrexate	1
Mycophenolate mofetil	1
Intravenous immunoglobulin	2
Rituximab	2
Abatacept	1
Tocilizumab	1

Level of evidence: 1, indicates randomized controlled trial; 2, uncontrolled trial.

### Methotrexate

Methotrexate is the most commonly administered immunosuppressive in patients with SSc and has been shown to be effective in multiple prospective trials. In one randomized controlled trial, 29 SSc patients with early progressive skin disease received either 15 mg methotrexate weekly (n = 17) or placebo (n = 12). After 24 weeks, total skin score (*P* = 0.06) and creatinine clearance rate (*P* = 0.07) were found to be reduced in the patients who received methotrexate compared with those who received placebo whereas visual analogue of general well-being score (VAS) increased (*P* = 0.19)^6^. In a subsequent multicenter randomized controlled trial, early-onset (<3 years) SSc was treated with either 15 mg MTX weekly (n = 35) or placebo (n = 36). Modified Rodnan skin scores (mRSSs) for MTX-treated patients began to improve after 3 months of treatment (25.8 ± 2.9) compared with placebo-treated patients (28.8 ± 2.1) and persisted after 12 months (21.4 ± 2.8 and 26.3 ± 2.1, respectively)^7^. Despite the improvement in skin disease, no significant effect on interstitial lung disease (ILD) was noted. Most recently, the European prospective observational cohort found that patients taking methotrexate (n = 65), mycophenolate mofetil (MMF) (n = 118), or cyclophosphamide (n = 87) all had statistically significant improvement in their mRSSs and there was no significant difference between treatments (−4, −4.1, and −3.3, respectively)^8^. The European League Against Rheumatism******** currently recommends methotrexate as the first-line therapeutic for treating skin involvement in early progressive skin disease^5^. However, given the lack of evidence for efficacy in ILD, methotrexate should be used in those without ILD. Given the efficacy of MTX in inflammatory arthritis and dermatomyositis, it is a good choice for patients who may have concomitant features of these conditions.

### Mycophenolate mofetil

The use of MMF in treating SSc is supported by several small observational studies. In one study, 69 patients who received MMF (titrated to 3 grams per day) in the Scleroderma Lung Study II were compared with 79 patients in the placebo arm of the Scleroderma Lung Study I^9^. In this study, the cohort of patients had early progressive skin disease and those who received MMF had significantly improved percentage predicted forced vital capacity (FVC) (*P* <0.0001), percentage predicted hemoglobin‐adjusted single‐breath diffusing capacity for carbon monoxide (DL_CO_) (*P* <0.0001), and dyspnea (*P* = 0.0112) compared with patients who received placebo over 2 years. mRSS was a secondary outcome measure, and patients who received MMF had decreased mRSSs compared with patients who received placebo (10 versus 13, respectively; *P* <0.0001). A previous study investigated intrinsic gene expression subsets of SSc skin. Of 7 patients who received MMF, 4 had improvement in their mRSSs. All 4 of these patients were determined to have inflammatory intrinsic subsets, whereas the 3 non-responders had normal-like or fibroproliferative intrinsic subsets^10^. MMF is a preferred option for treatment of SSc-related skin disease, particularly for patients with ILD or those with progressive skin disease who are unable to tolerate methotrexate.

### Intravenous immunoglobulins

Intravenous immunoglobulin (IVIG) is used to treat a host of autoimmune diseases and its use in patients with SSc was first reported in 2000 in three patients with rapidly progressing skin disease^11^. Many studies that have investigated the use of IVIG in treating SSc are small uncontrolled studies reporting different IVIG doses and infusion schedules (1–2 g/kg of body weight, administered over 2–5 days). One of the largest studies to date involved 30 dcSSc patients who were on concomitant immunosuppressives with refractory disease^12^. They were noted to have improvement in skin thickening at 12 months compared with historical controls from negative clinical trials. In one case report, a patient received IVIG (400 mg/kg per day for 5 days) with repeated courses every 10 days and had significant improvement in muscular performance and pulmonary function tests^13^. In patients with features of dermatomyositis, for which IVIG is a mainstay of treatment, IVIG may be a preferred second-line therapeutic option.

### Rituximab

Rituximab is a chimeric monoclonal antibody that targets CD20, a B-lymphocyte surface molecule that assists in differentiation and development of B cells into plasma cells. Its use in the treatment of SSc is of interest as abnormalities in B-cell function in SSc are thought to play a role in disease pathology. B-cell infiltrates have been demonstrated in both the skin and lung specimens in SSc^10,14,15^. In a case-control study conducted with patients enrolled in the European Scleroderma Trial and Research (EUSTAR) cohort, 63 patients with the diffuse cutaneous subtype received one course of rituximab and their mRSSs were compared with 25 patients with untreated SSc^16^. After 7 months, the mRSSs had decreased from 18.1 to 14.4 (*P* = 0.0002). When only patients with severe diffuse disease were analyzed (n = 25), mRSSs decreased from 26.6 to 20.3 (*P* = 0.03). Secondary analyses studied the effects of rituximab on lung fibrosis, and patients who received rituximab had a 0.4% increase in FVC whereas patients who received placebo had a 7.7% decrease. These studies suggest that rituximab may be a promising second-line therapy, especially for patients with skin and lung disease.

### UVA-1 phototherapy

UVA-1 (340–400 nm) phototherapy has been used in various T cell–mediated diseases, such as atopic dermatitis and mycosis fungoides, as it is thought to penetrate deep into the dermis to induce apoptosis of infiltrating T cells. Although the efficacy of UVA-1 phototherapy in SSc has not been extensively evaluated, it is hypothesized to have therapeutic value given the infiltrating T lymphocytes present in SSc-affected skin and internal organs^17–19^. Additionally, UVA-1 irradiation has been demonstrated to upregulate matrix metalloproteinases, leading to collagen breakdown^20–22^. The few studies that have been conducted have focused primarily on the treatment of affected hands and not the entire body in patients of unknown SSc subtype or disease duration. One open pilot study found that lower-medium-dose (30 J/cm^2^) UVA-1 phototherapy improved acrosclerotic lesions, and 7 out of 8 patients reported marked improvements in finger motion, skin elasticity, and ulcerations^23^. A subsequent open non-randomized study included 18 dcSSc patients who received low-dose UVA-1 phototherapy (30 J/cm^2^)^24^. Of the 18 patients, 16 had clinical improvement with marked softening of the previously affected skin upon palpation and inspection as well as improvement in finger mobility. Six months after treatment, the majority of patients had stable clinical outcomes. Another study administered low-dose UVA-1 phototherapy (40 J/cm^2^) three times weekly to 9 patients with SSc^25^. The dorsal and palmar surfaces of one hand were irradiated while the other hand served as a control. After 14 weeks, mean clinical scores of treated hands decreased significantly (*P* <0.05). However, there were no significant differences between the treated and untreated hands^25^. Importantly, the effect of UVA-1 on SSc skin involvement outside the hands and in patients with early inflammatory disease has not been examined systematically. Some retrospective reviews indicate that UVA-1 phototherapy may be of benefit in this patient group. Optimum doses and protocols for UVA-1 phototherapy have yet to be determined as data on the duration of treatment for specific subsets of SSc are lacking. Nonetheless, clinicians may consider UVA-1 phototherapy as adjuvant therapy for patients with early diffuse cutaneous disease as either an adjunct or first line if there are contraindications to systemic immunosuppressives.

### Other emerging therapies

The use of tocilizumab, an interleukin 6 receptor alpha inhibitor, has previously been studied, and phase III studies are under way. In the phase II trials, 43 patients with progressive SSc received 162 mg weekly tocilizumab subcutaneous injections and were compared with 44 patients who received placebo^26^. Although there was not a significant reduction in skin thickening as assessed by mRSSs in patients who received tocilizumab, the degree of change was greater than that of the placebo group at 24 weeks. Furthermore, patients in this group had less decline in FVC. These promising results suggest that tocilizumab is a potential therapeutic for patients in the future.********

Additionally, previous studies have provided evidence that T cells may play a significant role in the pathogenesis of diffuse SSc, especially its cutaneous manifestations. On skin biopsy samples, T-cell infiltrate has been noted, and the amount of T-cell lymphocytes present has been found to correlate with the degree of skin thickening^17–19^. As such, abatacept, a medication that interferes with the activation of T cells, is thought to be a promising therapeutic for the treatment of SSc. In a phase II trial, 34 patients with diffuse SSc received 125 mg abatacept subcutaneous injections and were compared with 35 patients who received placebo^27^. Although primary outcome measures (mRSS) did not differ significantly between the abatacept and placebo groups, there were statistically significant treatment differences in the Health Assessment Questionnaire Disability Index at 12 months.

### Selection of treatment

**Figure 2.**
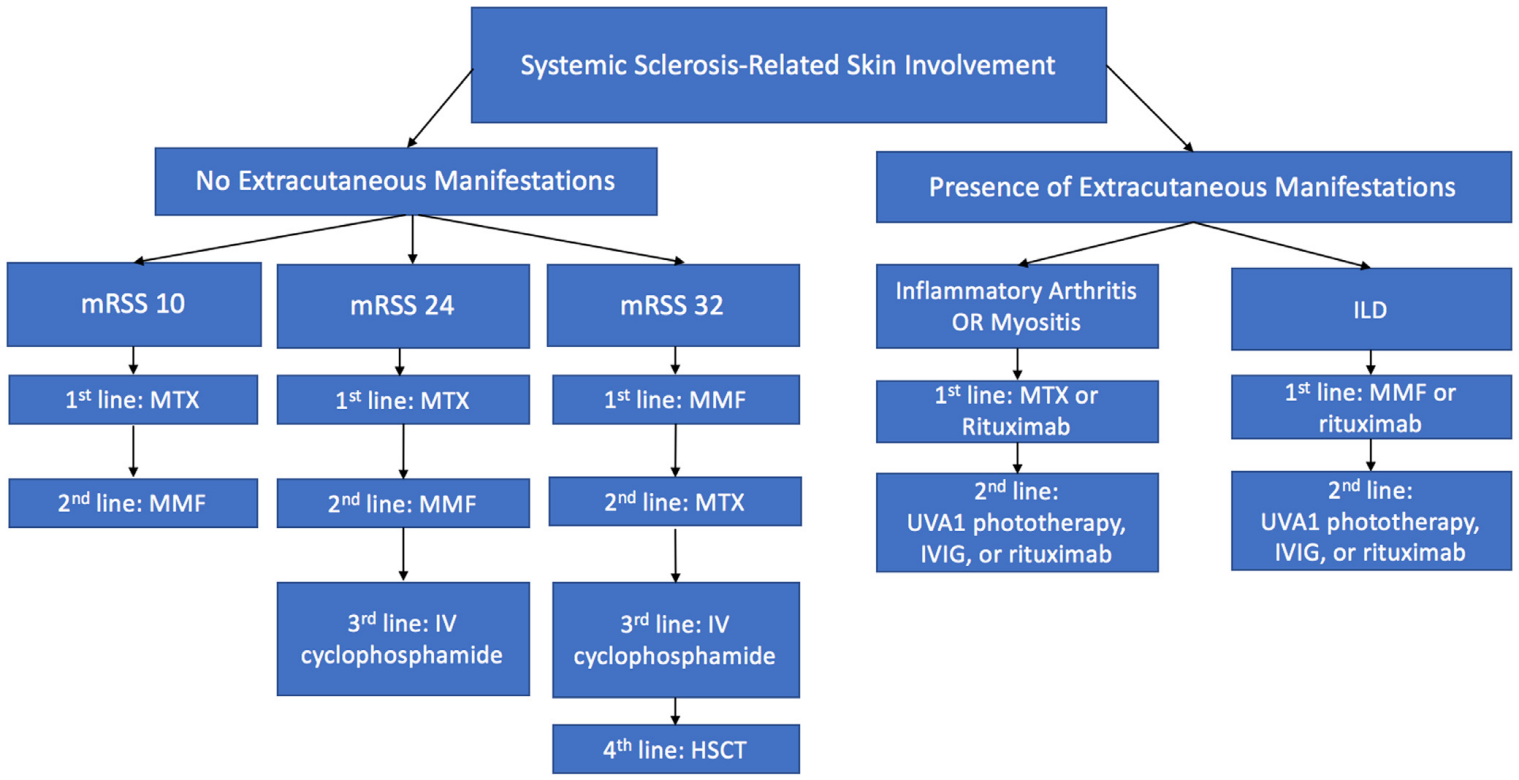
Therapeutic algorithm for scleroderma based on existing evidence. Abbreviations: HSCT, hematopoietic stem cell transplantation; ILD, interstitial lung disease; IV, intravenous; IVIG, intravenous immunoglobulin; MMF, mycophenolate mofetil; mRSS, modified Rodnan skins core; MTX, methotrexate.

The authors recommend using an evidence-based algorithm when considering therapeutic options for patients with SSc (Figure 2). The approach to the patient with SSc depends on the assessment of disease subtype and the presence of other organ complications in addition to cutaneous involvement^28^. Importantly, patients with early-onset diffuse skin manifestations respond best to treatment. In those with diffuse progressive skin disease and inflammatory arthritis, methotrexate or rituximab monotherapy should be used as first-line therapy. Methotrexate or IVIG should be used in patients who have dcSSc with concomitant myositis but without substantial ILD. In patients with skin disease and ILD, studies have suggested that MMF or rituximab may have the best efficacy. If patients fail these first-line therapies, providers should consider adding second-line treatments, including UVA-1 phototherapy, IVIG, or rituximab. Lastly, in patients with solely cutaneous manifestations, treatment should be dictated by disease severity as measured by mRSSs after careful assessment for internal organ involvement (Figure 2).

## Treatment of other cutaneous manifestations of systemic sclerosis

### Treatment of Raynaud’s phenomenon

Raynaud’s phenomenon frequently occurs in patients with SSc and is due to pathologic effects on small blood vessels. The initial treatment approach is lifestyle modification to avoid activities that exacerbate vasoconstriction, including smoking and exposure to cold. However, given the secondary nature of this phenomenon, pharmacologic treatment is often warranted. Treatment is firstly guided on the basis of disease acuity. Many patients may have chronic, non-critical digit ischemia that poses minimal risk of digit loss. However, critical digit ischemia, characterized by an exquisitely painful, cyanotic digit, is worrisome for development of dry or gangrenous necrosis. Any patient presenting with signs of critical digit ischemia should notify their provider immediately for hospital admission. The patient should receive anticoagulant therapy and treatment for any overlying infection. Workup for thrombotic/embolic complications is imperative. If these complications are present, IV Flolan, Remodulin, and Veletri (IV prostanoid) are digit-salvaging medications that should be considered. These are important considerations in complicated Raynaud’s in which saving the affected digit is paramount. However, owing to frequent side effects, patients with uncomplicated Raynaud’s should not receive the aforementioned medications. Instead, pharmacotherapy for chronic ischemia from Raynaud’s is aimed at improving pain, preventing ulcer formation, and enhancing wound healing. Importantly, the characteristic change in digit coloration (from white to blue to red) will likely not improve with treatment. Treatment should be initiated or escalated when fingertip ulcerations are present as this indicates poorly controlled Raynaud’s. First-line treatments include nitrates or calcium channel blockers (CCBs). Amlodipine 5 to 20 mg daily can be safely used at a 5-mg starting dose with updosing as needed. Of note, clinical trials studying the effects of these therapies delineate effect differences; some prevent ulcer development whereas others promote wound healing. CCBs for treatment of Raynaud’s were evaluated in a systematic review and meta-analysis of 23 clinical trials involving 528 patients. CCBs reduced both vasoconstrictive attack severity and average number of weekly attacks by six when compared with placebo^29,30^. Importantly, oral CCBs aim to prevent ulcer development whereas topical CCBs promote wound healing. Among other first-line therapies of consideration are phosphodiesterase 5 (PDE5) inhibitors. A meta-analysis of double-blind clinical trials of PDE5 inhibitors in secondary Raynaud’s found this drug to have moderate efficacy in reducing the frequency of daily ischemic attacks. Alternatively, if CCBs are contraindicated, angiotensin receptor blockers, serotonin reuptake inhibitors, and endothelin receptor antagonists such as bosentan can be helpful, although data are limited. Thus far, studies evaluating the effect of angiotensin-converting enzyme inhibitors on Raynaud’s have been inconclusive^29,31,32^. Regardless of treatment selection, all patients should avoid medications that promote vasoconstriction, such as nasal decongestants and amphetamines.

### Treatment of calcinosis cutis

Calcinosis cutis is commonly associated with long-standing SSc and may complicate both limited and diffuse cutaneous disease. It is characterized by calcium deposits in the skin and subcutaneous tissues. Although no treatments are widely accepted as standard therapy, many have been reported to be beneficial in the literature. For smaller calcium deposits, warfarin^33^, surgical excision, carbon dioxide laser^34^, ceftriaxone^35^, and IVIG^36^ have been used. In contrast, larger calcified lesions may require curettage, surgical excision, probenecid, diltiazem^37^, aluminum hydroxide^38^, or bisphosphonates^39^.

### Treatment of microstomia

Patients with SSc frequently have decreased oral opening or microstomia as collagen deposits lead to fibrosis in perioral tissue. This leads to decreased range of motion of the mandible, impeding mastication, speech, and oral hygiene. Current treatment recommendations include mouth-stretching and oral augmentation exercises^40^. Recent case reports indicate that botulinum toxin or hyaluronidase injected periorally may be of benefit^41^.

### Treatment of telangiectasias

In patients with SSc, cutaneous telangiectasias can develop, most commonly several years after disease onset, because of dilation of microvessels and can be a source of psychological distress. Current treatment recommendations include camouflage techniques such as specialized makeup, pulse dye laser (PDL), and intense pulse light (IPL). In one study, 19 patients with telangiectasias received three treatments with PDL on one side and IPL on the other side^42^. The primary outcome measure was the difference in mean improvement in photographic appearance of telangiectasias at week 16. Lesions treated with IPL and PDL both improved at week 16 from baseline. However, comparison between PDL and IPL revealed a greater improvement with PDL than IPL (*P* = 0.01). Patients reported transient bruising after treatment with PDL, but no side effects were reported for IPL^42^.

## General skin care in systemic sclerosis

Patients with SSc have a predisposition to develop a pruritic rash, such as asteatotic eczema and hand dermatitis. Furthermore, up to 42.6% of patients with SSc report pruritus as a bothersome symptom of disease^43^. Thus, a gentle skin-care regimen is of utmost importance for preventing and treating these conditions. A patient should be advised to limit baths and showers to 10 to 15 minutes. They should use lukewarm water and minimize the use of soap, especially in dry areas of the body. A patient may consider soap only in the axillae, groin, and feet. Afterwards, care should be taken to pat the skin dry instead of rubbing. Additionally, moisturizers maintain the integrity of the stratum corneum, the top layer of the epidermis^44^. Thus, patients should apply moisturizers 2 to 3 minutes after drying. Thicker, fragrance-free moisturizers, including ointments and petroleum jelly, are most effective at retaining skin moisture. Choice of emollient should be guided by patient preference.

## Summary and conclusions

SSc is a connective tissue disease that leads to progressive fibrosis of the skin and internal organs. Treatment of scleroderma continues to be a challenge as targeted therapies are lacking. Although methotrexate remains the most commonly used therapy in treatment of scleroderma-related cutaneous disease, studies investigating the utility and efficacy of other therapeutics, including MMF, IVIG, and UVA-1 phototherapy, are promising. When treating scleroderma, clinicians should consider the patient’s specific organ complications to guide therapeutic selection and management.
